# Proton‐Coupled Electron Transfer in Cytochrome *c* Oxidase: Heme *a* Controls the Protonation Dynamics of E286

**DOI:** 10.1002/cphc.202500539

**Published:** 2025-11-08

**Authors:** Federico Baserga, Pit Langner, Luiz Schubert, Julian P. Storm, Ramona Schlesinger, Joachim Heberle

**Affiliations:** ^1^ Department of Physics Experimental Molecular Biophysics Freie Universität Berlin Arnimallee 14 14195 Berlin Germany; ^2^ Department of Physics Genetic Biophysics Freie Universität Berlin Arnimallee 14 14195 Berlin Germany

**Keywords:** electron transfers, oxidoreductases, photolysis, protonation, time‐resolved spectroscopy

## Abstract

Complex IV of the mitochondrial respiratory chain, or cytochrome *c* oxidase (C*c*O), contributes to the proton motive force necessary for ATP synthesis. C*c*O can slow the formation of reactive oxygen species and is key to physiology and drug development. The exact molecular mechanisms underlying its proton‐pumping function remain elusive. The redox state of C*c*O's metallic cofactors is intimately connected to structural changes and proton pumping via proton‐coupled electron transfer. Time‐resolved UV/Vis and IR spectroscopy are used to investigate the effects of the electronic backreaction triggered by photolyzing the CO‐inhibited 2‐electron reduced state, **R**_**2**_ **CO**, in the *aa*_3_ oxidase from *Cereibacter sphaeroides*. An intermediate is identified, in which the binuclear center matches the redox state of the catalytic intermediate **E** (one‐electron reduced state), with a rise time of ≈2 μs. The electron transfer induces structural changes that lead to E286 deprotonation, with a time constant of 13 μs. Thus, it is inferred that transient reduction of heme *a* alone drives E286 deprotonation. E286 is reprotonated with a time constant of 72 ms when CO rebinds. The results support the view that transient heme *a* reduction in the physiological **E** state modulates the electrostatic environment, triggering proton transfer toward the proton‐loading site.

## Graphical abstract

Deprotonation of the glutamic acid 286 in cytochrome *c* oxidase from *Cereibacter sphaeroides* occurs 13 μs after photolysis of the CO ligand. The reaction is triggered by subsequent backward electron transfer from the heme *a*_3_ to heme *a*, which immediately precedes the deprotonation event. Therefore, transient reduction of heme a during the physiological forward reaction controls the proton‐coupled electron transfer mechanism.

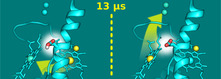

## 1 Introduction

Given its central role in the aerobic respiratory chain, cytochrome *c* oxidase (C*c*O) has been studied extensively in the last decades. It was discovered that its main function is to pump protons from the matrix to the intermembrane space^[^ ^1^ ^]^ (the membrane being mitochondrial or bacterial), thus contributing to the proton motive force that drives chemiosmosis.^[^ ^2^ ^]^

In its native environment, C*c*O undergoes a continuous cycle of electron injections from reduced cytochrome *c* (cyt*c*), which are exploited as an energy source when they combine with molecular oxygen and protons.^[^ ^3^ ^]^ The large energy gap between the redox couples O_2_/H_2_O and cyt*c* ^2+^/cyt*c* ^3+^, reaching roughly 51 kcal mol^−1^ over the whole cycle,^[^ ^4^ ^]^ is used to reduce molecular O_2_ and to pump protons against the membrane gradient.^[^ ^5^ ^]^ This series of reactions represents a hallmark in the field of proton‐coupled electron transfer (PCET),^[^ ^5^ ^]^ which extends from relatively simple inorganic redox‐active molecules to complex systems exhibiting multistep concerted processes involving several electrons and protons at the same time, such as those involved in photosynthesis.^[^ ^6^ ^]^

**Figure 1:**
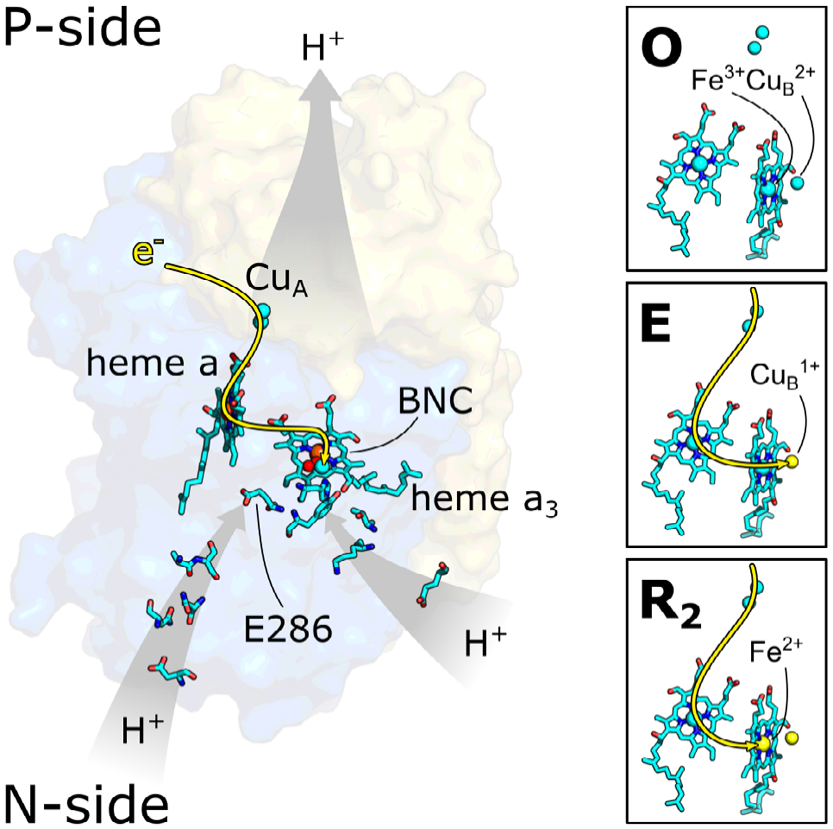
Schematic representation of PCET in *Cs*C*c*O (PDB ID: 2GSM). Protons are uptaken through two Grotthus channels facing the N‐side of the membrane (K and D pathways). The electron tunneling pathway is highlighted in yellow. Insets illustrate the redox states of the metal cofactors in the BNC during the first three intermediates of the catalytic cycle (yellow: Reduced; cyan: Oxidized).

In vivo, the catalytic cycle of C*c*O entails a series of redox changes that are initiated by the sequential injection of four electrons. These changes involve the cofactors Cu_A_, heme *a*, heme *a*_3_ and Cu_B_.^[^ ^7^ ^]^ The latter two form the so‐called binuclear center, or BNC, in which water formation takes place.^[^ ^8^ ^]^ The residues in the vicinity of the cofactors are of major interest since they couple proton and electron transfer. During a full catalytic cycle, two electrons are first injected, resulting in the one‐ and two‐electron reduced states, **E** and **R**_**2**_, in which first Cu_B_ and subsequently heme *a*_3_ are reduced to their 1+ and 2+ oxidation states (see **Figure** **1**). After the **R**_**2**_ intermediate is formed, O_2_ can bind to the BNC and two additional electrons can be accepted; each electron transfer is coupled to a pumped proton, with an additional proton uptaken for charge compensation and water formation.^[^ ^9^ ^]^ Therefore, the chemistry of the BNC itself, which is still not completely elucidated, is at the heart of research targeting C*c*O. In particular, the sequence of molecular events is yet to be assessed, because the full catalytic reaction of the enzyme cannot be tracked by time‐resolved experimentation: currently, there are no protocols for triggering and synchronizing an ensemble of C*c*O molecules during sequential electron injection by its natural substrate. Stabilization or trapping may lead to intermediate states that are not necessarily relevant to the physiological reaction.^[^ ^10^, ^11^ ^]^ Newly published resonance Raman data suggested a peroxide ligand at the BNC in the resting state of the enzyme,^[^ ^12^ ^]^ and a revised cycle has been recently proposed on the basis of cryogenic electron microscopy.^[^ ^13^ ^]^ It has even been suggested that structural gating plays a role in the proton‐pumping reaction.^[^ ^14^ ^]^

While it is difficult to induce the physiological reaction of C*c*O and quantitatively monitor it using spectroscopic techniques, time‐resolved information on the catalytic cycle of the protein can be retrieved by simplifying the system. One way to do so is to use smaller and less complex enzymes as a model organism, like bacterial oxidases such as the aa_3_ enzymes from *Paracoccus denitrificans* or *Cereibacter sphaeroides* (*Cs*C*c*O).^[^ ^15^, ^16^ ^]^ These bacterial enzymes lack the additional subunits present in the mammalian complex but share a very high degree of homology in the first two core subunits. Another route to simplification is to poise certain intermediate states of the enzyme and induce a nonphysiological reaction which mimics the reductive half of the catalytic cycle. Ligation with carbon monoxide (CO) is often exploited to generate a photolabile state.^[^ ^17^ ^]^ The experimental methods traditionally use transient UV/Vis spectroscopy to monitor either the rebinding of the CO ligand to heme *a*_3_ (flash photolysis) or the reaction of the fully reduced enzyme with molecular O_2_ (stopped‐flow photolysis) upon photolysis of the CO ligand.^[^ ^18^ ^]^

A surrogate reaction that has often been monitored is the photolysis of the mixed‐valence state (**R**_**2**_ **CO**), in which the BNC binds CO. The **R**_**2**_ **CO** state is peculiar in its electronic structure, with heme *a*_3_ and Cu_B_ at the BNC being reduced by 2e^−^, but heme *a* and Cu_A_ remain in their oxidized states.^[^ ^19^ ^]^ This process has been monitored thoroughly by visible absorption spectroscopy,^[^ ^17^, ^20^, ^21^, ^22^, ^23^, ^24^, ^25^, ^26^, ^27^ ^]^ and it induces an electron backflow reaction whose general mechanism under anoxic conditions is well understood: initially, the CO ligand gets photolyzed from **R**_**2**_ **CO**, and the central iron of heme *a*_3_ remains in its ferrous form; then, the electronic density shifts towards heme *a*,^[^ ^23^ ^]^ creating a state where heme *a* and Cu_B_ are reduced (but the other cofactors are oxidized).^[^ ^26^ ^]^ Regarding the redox state of the BNC, this is thought to be analogous to the **E** state of the catalytic cycle, from which the second proton is pumped upon the injection of a second electron.^[^ ^4^, ^28^, ^29^ ^]^ Electron backflow towards Cu_A_ follows, and this initiates the electron forward reaction. The last, slow phase simply represents CO rebinding to the BNC. Even though visible spectroscopy greatly contributed to the understanding of this reaction, it cannot deliver information involving the state of singular amino acid side chains. The chemical details reside in understanding the orchestrated PCET involving the redox cofactors and protonatable residues by which they are gated. The emerging electric fields play a fundamental role as these influence polarizable groups as well and finally trigger structural changes of the protein.^[^ ^30^ ^]^

Time‐resolved infrared spectroscopy is a powerful tool to compare the electronic changes recorded by visible spectroscopy with single amino acid protonation events, as well as with conformational or cofactor‐related structural changes.^[^ ^31^ ^]^ This technique is particularly valuable for studying photo‐induced reactions, whether naturally occurring or artificial, since the molecular ensemble can be synchronized through a short laser pulse, enabling the precise observation of the ensuing dynamic processes.^[^ ^32^ ^]^ The advent of state‐of‐the‐art tunable quantum cascade laser (QCL) heads allows us to follow small transient differences in the IR absorption of proteins at ns time resolution.^[^ ^33^ ^]^ The resulting kinetics can be mapped at different wavenumbers if the reaction being monitored is repetitive, or single‐shot kinetics can be achieved for nonrepetitive samples.^[^ ^34^ ^]^ We monitored the reaction resulting from photolysis of the **R**_**2**_ **CO** state of *Cs*C*c*O by scanning our QCLs in the frequency range of carboxylic acids vibration (1680–1770 cm^−1^) and in the range of CO vibration (1956–1972 cm^−1^). The spectrotemporal response was benchmarked using rapid‐scan Fourier‐transform infrared (FTIR) spectroscopy, which provides broad spectral data at the expense of poorer time resolution. By monitoring events from 100 ns onward with our QCL setup, we can resolve and distinguish the structural changes associated with electron backflow from the subsequent forward electron transfer, which reproduces the physiological transition from the intermediate state **E** to **R**_**2**_.

We observe a deprotonation reaction of the carboxylic acid side chain of glutamic acid 286 that correlates with the partial oxidation of heme *a*_3_ after photolysis of the CO ligand. Comparing this to the behavior of other vibrational bands and to the Soret bands at visible wavelengths, we conclude that, during the initial electron backflow, *Cs*C*c*O undergoes a “fast” reaction in which the reduction of heme *a* results in the deprotonation of E286, suggesting that the same mechanism is responsible for electric field reorientation^[^ ^30^ ^]^ and vectorial proton transfer^[^ ^35^ ^]^ during the transition from the oxidized state **O** to the physiological one‐electron reduced state, **E**, and the two‐electron reduced state, **R**_**2**_ (Figure 1).

## 2 Results and Discussion

Our experiments were designed to trace PCET in C*c*O by time‐resolved spectroscopy. Introducing CO as a photolabile ligand at the BNC facilitates the synchronization of the molecular reactions catalyzed by C*c*O. The rationale behind time‐resolved experiments is that the flash photolysis of fully reduced C*c*O provides information on ligand dissociation whereas, in the mixed‐valence state, CO photolysis induces electron back transfer from the BNC towards heme *a* and Cu_A_.^[^ ^20^, ^21^ ^]^ The electron transfer is monitored by UV/Vis spectroscopy and the proton transfer by IR spectroscopy: Both transient techniques cover the time range from 100 ns to 1 s.

*Cs*C*c*O was reconstituted in lipid nanodiscs as a biomimetic membrane that is considered to be closer to the cellular scenario than detergent or vesicular environments.^[^ ^36^ ^]^ Yet, IR spectra are influenced by the sample preparation protocol, which may differ between detergent‐solubilized samples and CcO reconstituted in nanodiscs (see Section 1 of the Supporting Information).

### 2.1 Electronic States and CO Rebinding Upon Photolysis of Mixed‐Valence C*c*O

**Figure 2:**
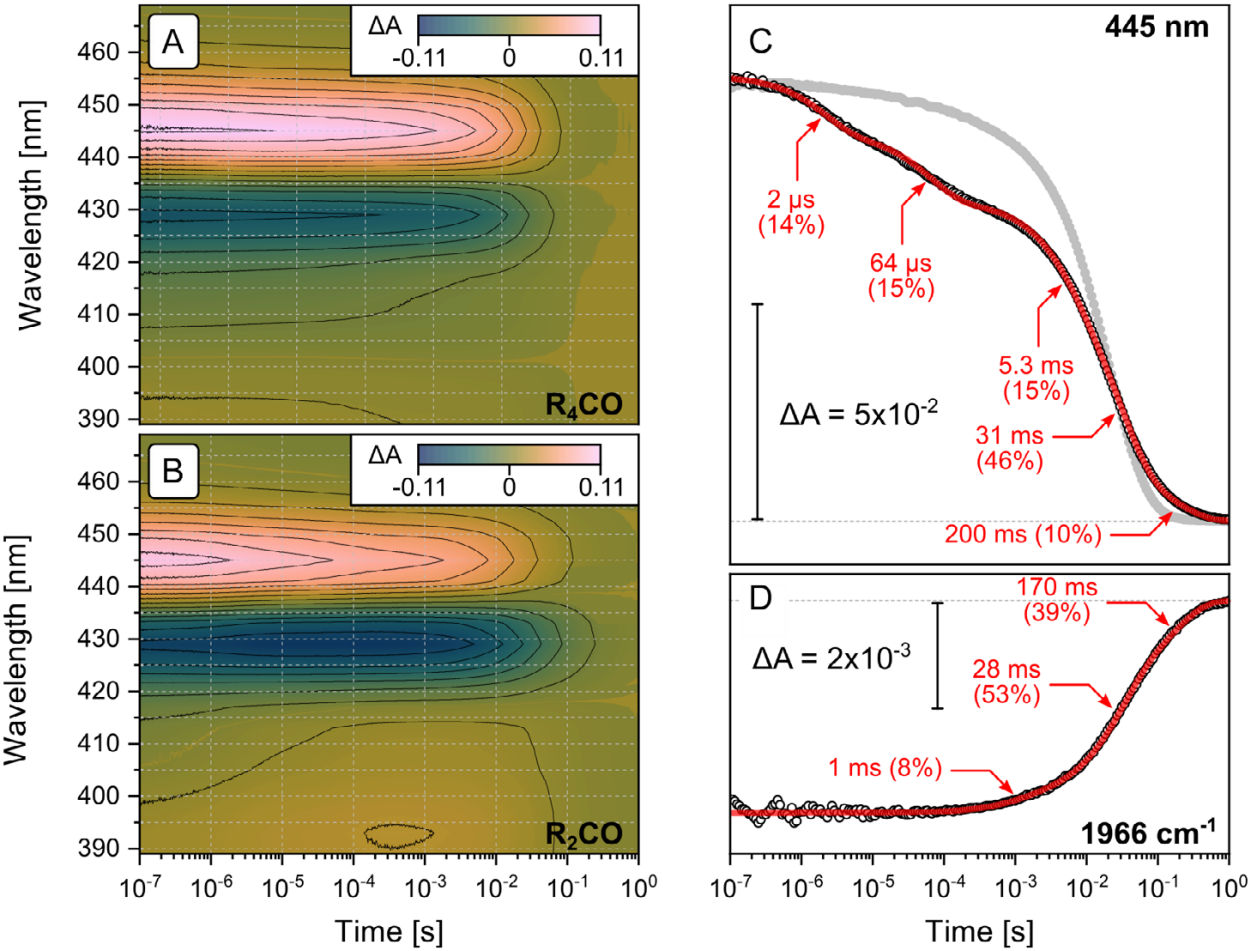
Cofactor changes upon photolysis of *Rs*C*c*O reconstituted in DPPC nanodiscs. A) Time‐resolved visible absorption changes initiated by photolysis of the **R**_**4**_ **CO** state. B) Time‐resolved visible absorption changes initiated by photolysis of the **R**_**2**_ **CO** state. C) Comparison between time‐resolved absorption changes of the **R**_**4**_ **CO** (gray circles) and **R**_**2**_ **CO** (empty circles) states monitored at 445 nm and the trace at 445 nm extracted from the global fit to the **R**_**2**_ **CO** dataset (red). D) Time‐resolved IR absorption kinetics of the *ν*(C≡O) band at 1966 cm^−1^ after photolysis of the **R**_**2**_ **CO** state (empty circles) and their fit to a sum of three exponentials (red). Time constants and relative amplitudes of the fits’ exponentials are indicated by red labels.

We recorded time‐resolved UV/Vis kinetics upon photolysis of the **R**_**2**_ **CO** state. Immediately after, the sample was reduced by the addition of Na_2_S_2_O_4_, inducing the **R**_**4**_ **CO** state. The time‐resolved kinetics of the **R**_**4**_ **CO** state upon CO photolysis were then measured. The mid‐IR kinetics after photolysis of the **R**_**2**_ **CO** state were recorded as well and compared with the UV/Vis data (see **Figure** **2**).

Flash photolysis of the fully reduced and CO‐bound *Cs*C*c*O (**R**_**4**_ **CO**) shows the depletion of its ground state at ≈430 nm and subsequent formation of the fully reduced **R**_**4**_ state which is characterized by the Soret band peaking at 445 nm (Figure 2A).^[^ ^20^ ^]^ This wavelength is representative of the reduced state of primarily heme *a*_3_. When all metal cofactors of C*c*O are in their reduced states, CO binds to Cu_B_ after the Fe—CO bond is broken and then dissociates from the BNC,^[^ ^37^ ^]^ to rebind in the ms time range and recover the initial state **R**_**4**_ **CO**.

Photolyzing the **R**_**2**_ **CO** state initially results in the same absorbance change at 445 nm as observed for **R**_**4**_ **CO** (Figure 2B). Subsequently, the band intensity decreases in multiple steps over time. The whole reaction completes in about 0.5 s under the applied conditions.^[^ ^24^ ^]^ We globally fit the kinetics shown in panel B of Figure 2 with the sum of five exponentials and obtained time constants of 2 and 64 μs for the initial phase of the reaction (Figure 2C). The fit provided time constants corresponding to 5.3, 31, and 200 ms for the slow phase of the reaction.

The stretching vibration of CO bound to the central Fe of heme *a*_3_ oscillates at a frequency of ≈1966 cm^−1^.^[^ ^38^, ^39^ ^]^ Tracking the kinetics of the *ν*(C≡O) vibrational band after photodissociation from the central Fe^2+^ (Figure 2D), we can directly observe ligand rebinding to heme *a*_3_ after photolysis of **R**_**2**_ **CO**. The time constants for this process are comparable to those observed in the “slow” phase of the kinetics observed at 445 nm. This observation supports the interpretation of the “slow” reaction phase as mainly correlating with CO rebinding.

### 2.2 Time‐Resolved Infrared Spectra

**Figure 3:**
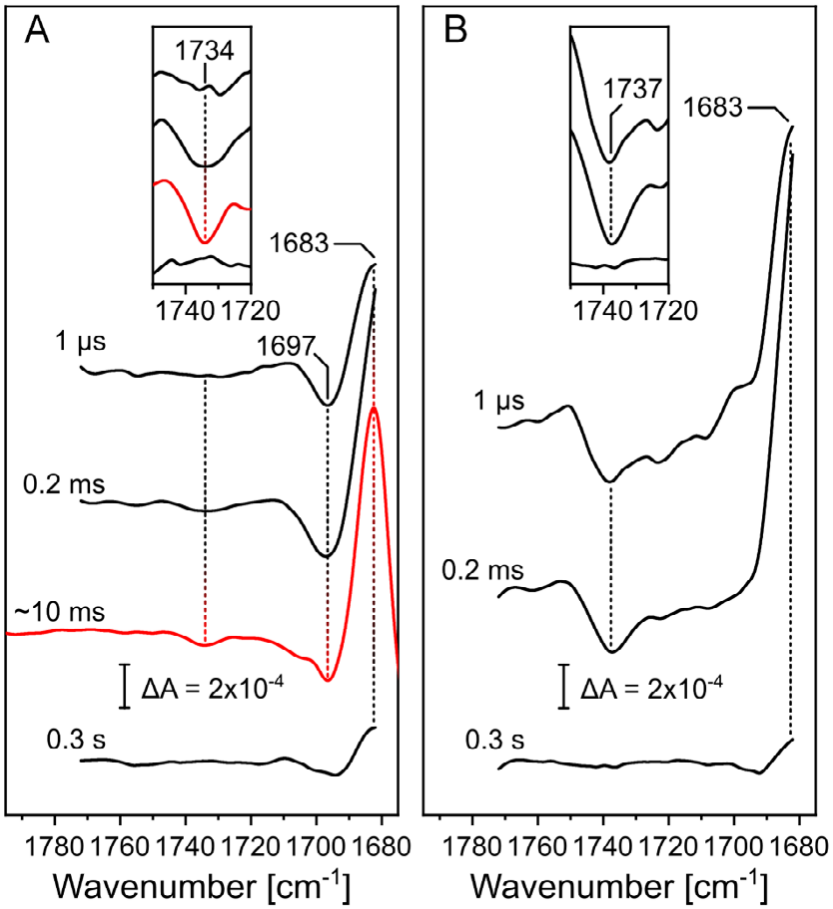
Time‐resolved IR absorption spectra upon photolysis of the **R**_**2**_ **CO** state. A) Time‐resolved difference spectra of *Cs*C*c*O reconstituted in ^13^C‐DPPC nanodiscs obtained from QCL mapping (black) and from rapid‐scan FTIR spectroscopy (red). Inset: Magnification of the 1720–1750 cm^−1^ range showing the carboxylic C=O stretching vibration of E286. B) Time‐resolved difference spectra of *Cs*C*c*O in ^12^C‐DPPC nanodiscs obtained from QCL mapping. Inset as in A). Bands are labeled by their respective frequencies and connected by vertical dashed lines.

The photolysis‐induced electron backflow can potentially induce structural changes in C*c*O. Therefore, we recorded time‐resolved IR differences in the 1680–1770 cm^−1^ range (see **Figure** **3**). This frequency range includes bands corresponding to the C=O stretching vibrations of carboxylic acid side chains from aspartic and glutamic acids, which are prime candidates for proton donors and acceptors in proteins. The negative band between 1750 and 1730 cm^−1^ (1745–1725 cm^−1^ in D_2_O) has been assigned to the deprotonation of the carboxylic acid moiety of E286 in *Cs*C*c*O.^[^ ^40^ ^]^ This residue plays a central role in determining the proton trajectory after uptake,^[^ ^35^, ^41^, ^42^ ^]^ and the kinetics of its vibrational band will be discussed below. We note that time‐resolved IR difference spectra associated with flash photolysis of the **R**_**2**_ **CO** state have previously been reported,^[^ ^43^, ^44^ ^]^ as well as steady‐state difference spectra under continuous illumination.^[^ ^45^ ^]^ However, these earlier studies lacked the temporal resolution necessary to isolate the phase associated solely with electron back‐transfer.

The C=O stretching vibration of the ester in the surrounding DPPC lipids absorbs at 1737 cm^−1^ in its weakly hydrogen‐bonded configuration and shifts by ≈40 cm^−1^ upon ^13^C‐isotope labeling.^[^ ^46^, ^47^, ^48^ ^]^ This band overlaps with the frequency range in which the band of the carboxylic C=O of the side chain of protonated E286 is expected to arise (Figure 3B). Thus, we monitored the CO‐photolysis reaction of *Cs*C*c*O reconstituted in ^13^C‐labeled DPPC to separate the contribution of the C=O stretch of the lipid ester, which absorbs at 1697 cm^−1^ for the lipid's isotopologue,^[^ ^46^ ^]^ leaving a weak band at 1734 cm^−1^ (Figure 3A). The band is observable in the ms time range, but absent in the early spectra at 1 μs, as well as in the late spectra at 0.3 s (see black spectra in the inset of Figure 3A). Since the same band is apparent in the rapid‐scan FTIR spectrum at 10 ms (red spectrum in the inset of Figure 3A), this feature is longer‐lived than 0.2 ms. The appearance of a single negative peak is indicative of the deprotonation of the E286 carboxylic acid side chain in *Cs*C*c*O.^[^ ^43^ ^]^

Finally, the most intense band in this frequency range appears at 1683 cm^−1^. Its intensity increases over time, reaching a maximum around 0.2 ms. A positive band at this frequency is consistently observed in the CO photolysis reaction,^[^ ^49^ ^]^ but the vibrational assignment is not clear and will be discussed in the next chapter.

### 2.3 Infrared Kinetics

The **R**_**2**_ **CO** state is metastable under ambient conditions and quickly turns into an over‐reduced species upon multiple laser excitations,^[^ ^43^ ^]^ similar to the photoreduction of the **O** state reported previously.^[^ ^50^ ^]^ However, the over‐reduced form is inactive in the probed IR regime, resulting only in a decrease of signal intensity (data not shown). To avoid ground‐state interconversion, care was taken to minimize excitation‐induced transitions. Given the limited number of available laser shots, a compromise must be made between acquiring kinetic data at different frequencies to enhance spectral coverage or performing additional coadditions at individual frequencies to improve signal‐to‐noise ratio.

**Figure 4:**
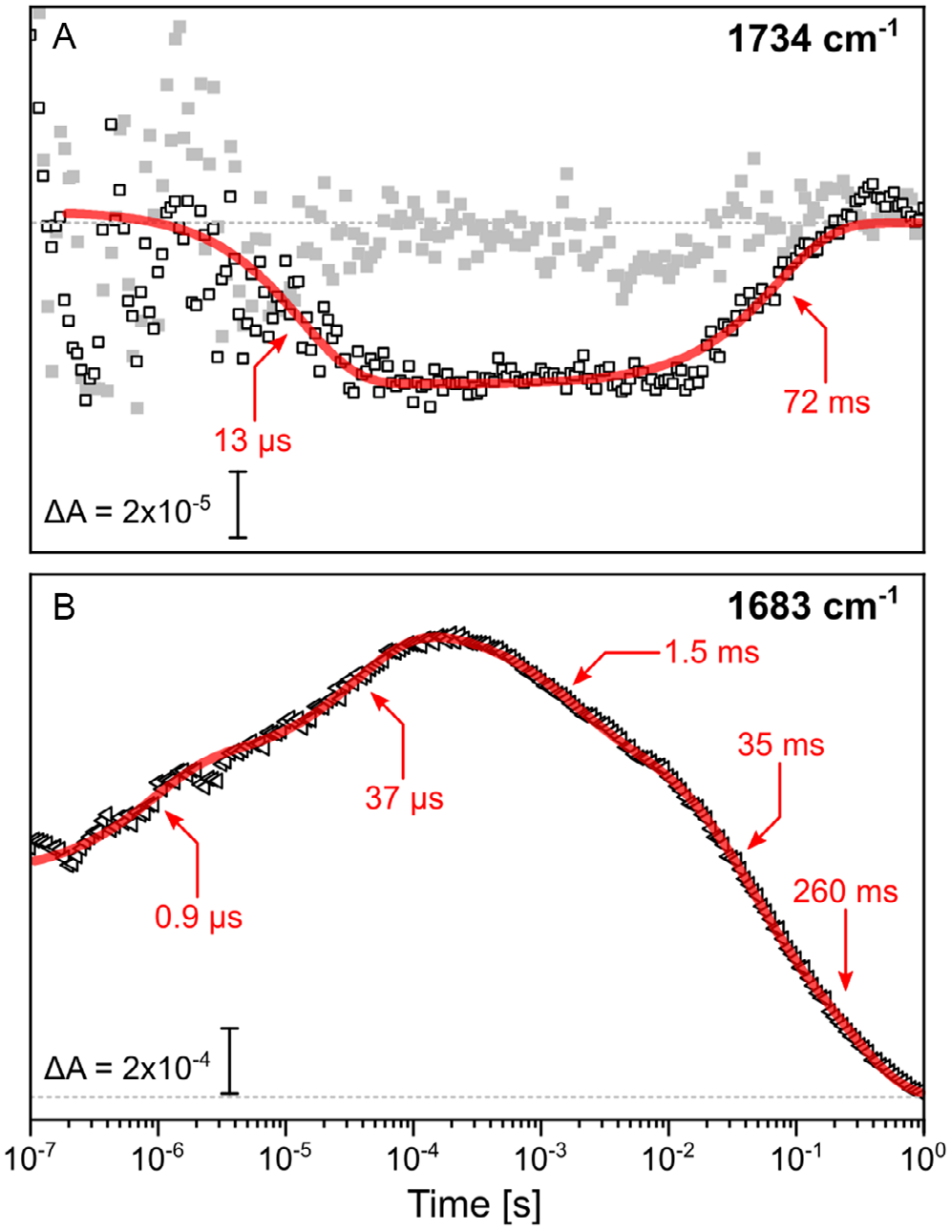
Time‐resolved IR absorption kinetics upon photolysis of the CO ligand. A) Kinetics obtained from single frequency acquisitions at 1734 cm^−1^ (empty squares) after photolysis of the **R**_**2**_ **CO** state. The data are corrected for the heating artifact of D_2_O as described in the supporting information and compared to the result of the same measurement upon photolysis of the **R**_**4**_ **CO** state (gray squares, subjected to identical data analysis). B) Single frequency acquisition kinetics at 1683 cm^−1^ (empty triangles) after photolysis of the **R**_**2**_ **CO** state. Multiexponential fits are shown as red lines. Time constants from the fits are indicated by red arrows.

We prepared a second sample from the same protein reconstitution batch using identical preparation procedures and measured selected kinetics at 1683, 1734, and 1742 cm^−1^ on the freshly prepared **R**_**2**_ **CO** state. To correct for spectral changes due to D_2_O heating by pulsed laser excitation, the kinetics at 1742 cm^−1^ were exponentially fit and subtracted from the raw data measured at 1734 and at 1683 cm^−1^ (data presented in **Figure** **4**, see also **Section 2** of the Supporting Information). As a control, we also induced photolysis of the **R**_**4**_ **CO** state on a different protein batch, measured kinetics at 1734 and 1742 cm^−1^, and corrected them in the same way.

The photolysis of the **R**_**2**_ **CO** and **R**_**4**_ **CO** states of *Cs*C*c*O monitored at 1734 cm^−1^ (Figure 4A) results in a transient negative signal for the former and only in noise for the latter. The transient absorption changes at 1734 cm^−1^ were fit by two exponential functions with time constants of 13 ± 6 μs and 72 ± 28 ms.

The difference band at 1683 cm^−1^ (Figure 4B) is ≈20 times more intense than the difference band at 1734 cm^−1^ and reflects more complex kinetics. The kinetics require the sum of five nonoffset exponential functions to be properly fit with time constants of 0.9 and 37 μs for the “fast” phase of the reaction, and values of 1.5, 35, and 260 ms for the “slow” phase.

The time constants derived from fitting the kinetics at 1683 cm^−1^ closely match those obtained from fitting the UV/Vis data (Figure 2C). This similarity suggests that the absorption arises from intermolecular cofactor *ν*(C=O) modes that respond directly to redox changes, rather than from amide I structural changes, which would require significant mass transport. Thus, we conclude that the band is associated with vibrations of the two heme cofactors, with the *ν*(C=O) stretching vibration of heme formyl as the prime candidate.^[^ ^51^, ^52^ ^]^ This assignment is consistent with the observed large change in absorption. A band at 1683 cm^−1^ has not yet been reported in resonance Raman spectra, possibly because of the transient nature of the **E** state. Assignments to *ν*(C=O) vibrations of the hemes’ protonated propionic acid side groups, of the protein backbone (amide I) in a *β*‐sheet configuration,^[^ ^53^, ^54^ ^]^ or of amino‐acid side chains such as asparagine, glutamine, or arginine are less favorable based on the size of the band.

## 3 Conclusions

Given the current understanding of the electronic back‐reaction after photolysis of the **R**_**2**_ **CO** state in C*c*O,^[^ ^7^, ^20^, ^23^, ^24^, ^29^ ^]^ our data provide a clear picture. Time‐resolved UV/Vis and IR spectroscopy were used to monitor both electronic and amino acid changes, revealing that the reaction of the partially reduced enzyme upon CO photolysis consists of a fast and a slow phase.

**Figure 5:**
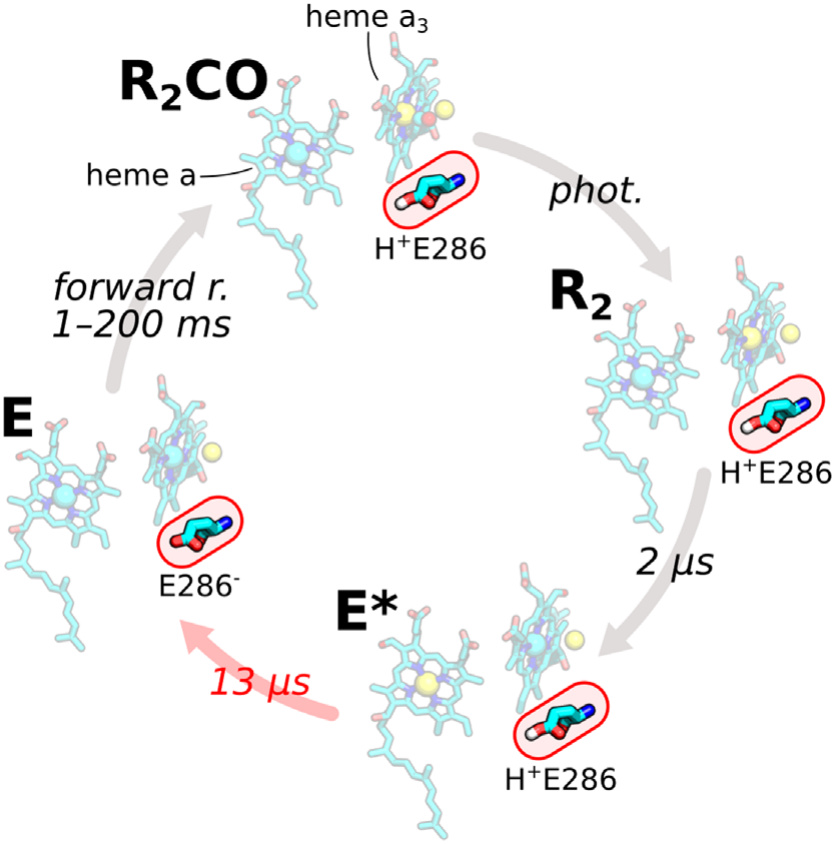
PCET in *Cs*C*c*O during the initial phase of the photolysis reaction. The scheme is based on the crystal structure of oxidized *Cs*C*c*O (PDB ID: 2GSM) and only intended as an explicative model for the observed reaction. The initial state is **R**_**2**_ **CO**, with the ligand artificially supplemented based on the fully‐reduced and CO‐bound bovine C*c*O (PDB ID: 3AG1). The ligandless mixed‐valence state obtained shortly after photolysis mimics the physiological **R**_**2**_ state. We designate the first intermediate state as E*, as it shares the same electron density at the BNC with the physiological **E** state. The electronic relaxation of **E** coincides with electron forward transfer and ligand rebinding, thereby mimicking the physiological **E **→ **R**_**2**_ transition. The metal ions of the cofactors are shown in yellow when they carry excess electrons compared to the **O** state.

**Figure** **5** illustrates the proposed reaction mechanism in the fast phase of the reaction, characterized by electron back‐transfer. The photolysis event itself is not resolvable with the time resolution of our experimental setup. Upon CO photodissociation, the *π*‐electron system of heme *a*_3_ responds immediately, and the first time constant (0.9–2 μs) reflects an initial shift in electronic density from the reduced BNC toward heme *a*,^[^ ^23^ ^]^ though a partial population of reduced heme *a*_3_ remains. In this intermediate, the BNC has the same electronic configuration as in the catalytic intermediate **E**, but electrons are still flowing in the backward direction. It is therefore named E*. The initial electron back‐transfer is also observable at 0.9 μs when indirectly measured through heme vibrational modes at 1683 cm^−1^. The deprotonation of E286 occurs at 13 μs, between the initial formation of E* (*τ* ≤ 2 μs) and the subsequent electronic shift toward Cu_A_ (*τ* ≈ 64 μs). This further electronic redistribution toward Cu_A_ is observable at 37 μs when monitored through heme vibrational modes at 1683 cm^−1^, too. These values time constants align well with those reported,^[^ ^23^, ^24^ ^]^ suggesting that nanodisc reconstitution does not significantly alter this fast reaction (unlike the reaction with O_2_^[^ ^55^ ^]^). The intermediate following E* and preceding CO rebinding at ≈1 ms (see Figure 2D) is considered analogous to the physiological **E** state.

The intermediates formed subsequently represent electron forward flow and mimic the physiological transition from the one‐electron‐reduced **E** state to the two‐electron‐reduced **R**_**2**_ state. During this slower phase, the BNC metal centers are initially in their Fe^3+^ and Cu_B_^1+^ states, and another electron tunnels from Cu_A_ towards the BNC,^[^ ^56^ ^]^ ultimately reducing heme *a*_3_ to Fe^2+^. Previous work^[^ ^43^ ^]^ demonstrated that this transition requires E286 to be deprotonated in the physiological **E** state of *Cs*C*c*O. By resolving the fast phase of the reaction, we gained the ability to monitor electronic backflow at a faster rate, enabling us to distinguish the contributions of heme *a* and heme *a*_3_. Ultimately, this allows us to correlate the transient reduction of heme *a* with the deprotonation of E286.

The deprotonation reaction of E286 produced a purely negative lineshape, with no indications of hydrogen‐bonding changes (Figure 3A). Its kinetics are described by only two processes: rapid deprotonation after the transient reduction of heme *a* and reprotonation concurring with the slow phase. Notably, the time evolution of intermediate “C” from the sequential scheme proposed by Szundi et al.^[^ ^24^ ^]^ closely matches the kinetics recorded at 1734 cm^−1^ (Figure 4A).

Our interpretation suggests that electron transfer between heme *a*_3_ and heme *a* does not immediately affect E286, whereas the subsequent oxidation of heme *a* creates the conditions for its deprotonation. During the forward reaction, heme *a* transiently reduces again as CO rebinds, destabilizing the metastable **E** state and leading to E286 reprotonation.

Finally, we observed a spectral response from the ester groups nanodisc lipids, with bands at 1697 and 1737 cm^−1^ for ^13^C‐DPPC and ^12^C‐DPPC, respectively. We previously reported a similar effect during the oxidation of nanodisc‐reconstituted *Cs*C*c*O and attributed it to a pressure‐induced lipid packing transition driven by increased protein volume in the oxidized state (**O**).^[^ ^47^ ^]^ Since our previous study lacked time resolution, its findings are not directly transferable.

While our results suggest that transient electron transfer to heme *a* drives deprotonation of E286 in the **E** state, they do not provide definitive evidence that the same heme group is involved in E286 reprotonation later in the catalytic cycle during forward electron transfer. Nevertheless, our data are consistent with this interpretation, possibly via orchestrated PCET involving E286 reprotonation through the D pathway.

In the native situation, electron injection from cyt*c* ^2+^ occurs twice during the reductive phase of the catalytic cycle (first to form **E** and then **R**_**2**_^[^ ^10^ ^]^) while heme *a* undergoes transient reduction during both events. Consequently, the transition from the **O** state, in which E286 is protonated,^[^ ^49^ ^]^ to the **E** state involves initial deprotonation of E286, which persists until the second electron injection transiently reduces heme *a*. Upon subsequent oxidation of heme *a*, i.e., before the formation of **R**_**2**_, E286 is reprotonated. It is noted that E286 is not identified as the residue responsible for proton release during formation of the **E** state, as this release reaction occurs later during the millisecond phase of the photolysis reaction.^[^ ^25^ ^]^ Recent studies align with this hypothesis, suggesting that BNC reduction triggers a protonation step that cannot take place via the D pathway.^[^ ^57^ ^]^

E286 is a key component of the molecular switch that prevents proton backflow during C*c*O's proton pumping mechanism, likely acting as a proton reservoir^[^ ^35^ ^]^ for the proton loading site. This residue is thought to reorient^[^ ^58^ ^]^ during different catalytic intermediates, facilitating proton transfer toward the proton‐loading site via a water‐mediated electric field.^[^ ^30^, ^42^ ^]^ This field is supposed to dictate the trajectory of protons uptaken from the D pathway, determining whether they contribute to proton pumping or chemical catalysis. If the protonation state of E286 is determined solely by the redox state of heme *a*, this implies that the first electron injection from cyt*c* ^2+^ could induce proton transfer from E286 to the propionate side chains of heme *a*_3_^[^ ^59^ ^]^ during the **O **→ **E** transition. In this model, heme *a*_3_ and residues adjacent to the BNC are the main drivers of chemical catalysis during the oxidative phase, while heme *a* functions as the “director” of the reaction during the reductive phase.

## 4 Experimental Section

4.1

4.1.1

##### Sample Preparation

*Cs*C*c*O was expressed, purified, and solubilized in detergent as described before.^[^ ^47^, ^60^, ^61^ ^]^ The detergent‐solubilized enzyme was then reconstituted in lipidic nanodiscs containing MSP1D1 as a scaffold protein^[^ ^62^ ^]^ and either dipalmitoylphosphatidylcholine (DPPC, Avanti, USA) or its ^13^C‐labelled isotopologue ^13^C‐DPPC (Cambridge Isotope Laboratories, USA). The detailed reconstitution protocol was published before.^[^ ^47^ ^]^ All data presented in the main text refer to samples in D_2_O buffer.

Liquid samples in a cuvette were prepared by removing gases dissolved in the buffer (Tris/DCl pH* 8.2, 5 mM), incubation with CO overnight,^[^ ^17^, ^19^ ^]^ and sealed to avoid oxygen diffusion before conducting the first set of measurements. Subsequently, the seal was removed, five equivalents of dry Na_2_S_2_O_4_ were added, followed by incubation with CO for 15 min before resealing the cuvette and conducting the second set of measurements.

Thin‐film samples sealed between two BaF_2_ windows were prepared similarly to before^[^ ^43^ ^]^ by exposing the sample (10 μl, ≈400 μM) to three vacuum/CO cycles in an anaerobic chamber connected to a Schlenk line. The cycles started with 20 s of vacuum, followed by 5 s of CO exposure for two times: the last cycle used 20 s of vacuum and 15 s of CO exposure before releasing the pressure. The pressure of CO and vacuum were +500 and −950 mbar relative to ambient atmosphere. The buffer used was Tris/DCl pH* 8.2, with a concentration of 5 mM before vacuum/CO cycles. Spacers of different thicknesses and materials were used for different experiments. A steady‐state FTIR spectrum in the frequency regime of 1950–1975 cm^−1^ was used to determine the successful preparation of the desired states (see Figure S3, Supporting Information).

An additional thin‐film sample was prepared following an almost identical protocol but depositing 5 μl of a D_2_O solution containing Na_2_S_2_O_4_ (20 mM) onto one of the two BaF_2_ window. The reducing solution corresponds to ≈10 eq of reductant for our C*c*O preparation and was dried under a gentle stream of N_2_ before depositing the sample (10 μl, ≈400 μM) on top of it and following with vacuum/CO cycles. This sample was used for control kinetics.

##### UV/Vis Spectroscopy

Time‐resolved UV/Vis measurements were performed using a LKS80 commercial flash photolysis setup (Applied Photophysics, UK) equipped with a Quanta‐Ray LAB 150 Nd:YAG laser (SpectraPhysics, DE). The samples were excited with the second harmonic (532 nm) of the Nd:YAG laser at 30 mJ cm^2^ energy density to photolyze the bound CO, triggering the reaction and data acquisition. Kinetics were recorded at 4 nm intervals, and two subsequent measurements with settings optimized for different time ranges were performed for each sample. The traces were merged before singular value decomposition analysis (SVD) was applied. Only SVD reconstructions of transient UV/Vis data are presented in the results section.

##### Infrared Spectroscopy

FTIR spectra were recorded using a Vertex 80v spectrometer (Bruker, DE) in rapid‐scan mode, in transmission configuration. Single‐sided interferograms were recorded after pulsed laser excitation at a spectral resolution of 4 cm^−1^ and a scanner velocity of 280 kHz, requiring ≈20 ms for each scan. The spectral window was electronically limited to 2250–0 cm^−1^, and a matching optical low‐pass filter was used. The sample was photoexcited at a repetition rate of 0.5 Hz, and a total of 5000 datasets were recorded and averaged to increase the signal‐to‐noise ratio. Difference absorbance was calculated using a spectrum averaged between ≈1.7 and ≈1.9 s after pulsed excitation, when the sample had decayed back to the **R**_**2**_ **CO** state.

IR kinetics with ns time resolution were recorded using a home‐built QCL spectrometer.^[^ ^33^ ^]^ To improve signal‐to‐noise ratio for the limited number of acquisitions, the oscilloscope measuring at high sampling rate was operated in AC‐coupled mode (using an analog high‐pass filter with a cutoff frequency of less than 12 Hz). The subsequent difference absorbance calculation was then based on the DC component extracted from the oscilloscope which recorded at a lower sampling rate. The laser excitation energy (at 532 nm) was kept at 20–30 mJ cm^2^ per pulse for the IR recordings. All data presented in the main text were recorded on samples at ambient temperature.

##### Data Analysis

Liquid H_2_O exhibits refractive index changes upon temperature variations, resulting in spectral changes observable in infrared measurements.^[^ ^63^, ^64^ ^]^ This is evident when monitoring the 1680–1770 cm^−1^ range of an essentially black‐body absorber upon 532 nm laser excitation (see Figure S4, Supporting Information). The thermal relaxation of the absorber (in our case, Na_2_S_2_O_4_‐reduced horse heart myoglobin) results in a broad negative band that is centered within the frequency range of interest.

Replacing H_2_O with D_2_O in the buffer partially resolves this issue, decreasing the intensity of the heating response and rendering it almost frequency‐independent (see Figure S4, Supporting Information). The residual response was subtracted from the IR time‐resolved kinetics presented in this work by using the kinetic at 1742 cm^−1^ as an internal reference for the D_2_O heating artifact, unless otherwise specified. The detailed procedure is described in Section 3 of the supporting information (refer to Figure S5, Supporting Information).

## Supporting Information

The authors have cited additional references within the Supporting Information.^[65–67]^

## Conflict of Interest

The authors declare no conflict of interest.

## Supporting information

Supplementary Material

## Data Availability

The data that support the findings of this study are available from the corresponding author upon reasonable request.
