# Supplementary material for: Proton‐Coupled Electron Transfer in Cytochrome c Oxidase: Heme a Controls the Protonation Dynamics of E286
Source: Chemphyschem. 2025 Nov 8;26(24):e202500539. doi: 10.1002/cphc.202500539 (PMC12710179; doi:10.1002/cphc.202500539)
Supplement: Supplementary file 1 — Supplementary Material [file CPHC-26-e202500539-s001.pdf]

Supporting information for:

Proton-Coupled Electron Transfer in Cytochrome *c* Oxidase: Heme *a* Controls the Protonation Dynamics of E286

Federico Baserga,+[a] Pit Langner,+[a] Luiz Schubert, [a] Julian P. Storm,[b] Ramona Schlesinger,[b] and Joachim Heberle*[a]

[a]
Department of Physics, Freie Universität Berlin
Experimental Molecular Biophysics
Arnimallee 14, 14195 Berlin, Germany
E-mail: joachim.heberle@fu-berlin.de

[b]
Department of Physics, Freie Universität Berlin
Genetic Biophysics
Arnimallee 14, 14195 Berlin, Germany

1. Effects of nanodiscs reconstitution

C*c*O samples show notable differences between their detergent-solubilized and nanodisc-reconstituted forms. Some of these differences are known to impact the activity of the enzyme, and are simply due to the presence of a membrane environment [1]. Other variations are often observed during the sample preparation steps: The high lipid-to-protein ratio typical of nanodiscs likely leads to a substantially increased local concentration of hydrophobic gases such as carbon monoxide, strongly influencing ligand affinity.

Figure S1 illustrates how the formation of the **R_2CO** by autoreduction [2, 3] occurs nearly 40 times faster when *Cs*C*c*O is reconstituted in nanodiscs containing polar lipids from *E. coli* compared to its detergent-solubilized form (Dodecyl-β-D-maltoside). This effect is pronounced in high-concentration preparations, such as those used for IR experiments, but remains less significant at low concentrations (i.e., in UV/Vis experiments).

The rapid CO autoreduction reaction in nanodiscs increases the likelihood of C*c*O overreduction. In fact, the fit of the CO binding kinetics for *Cs*C*c*O in nanodiscs (empty circles in **Figure S1**) reveals a transition from an initial exponential binding phase to a subsequent linear regime, indicative of the creation of an overreduced state [2].

Differences between detergent-solubilization and nanodisc-reconstitution also appear to influence the ground state of E286 in the **R₂CO** photolysis reaction.

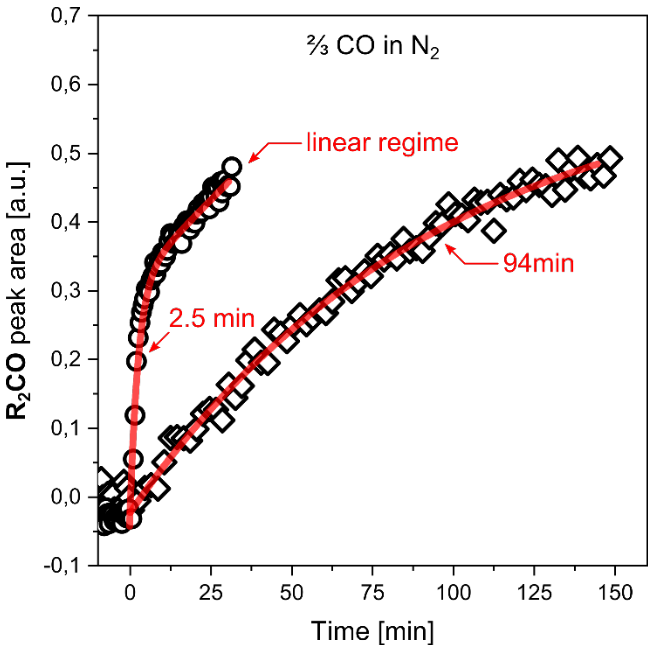

Figure S1. CO autoreduction of *Cs*C*c*O on ATR. Integrals of the Voigtian peak fit of the Fe-C≡O band during CO binding on the as-purified oxidized enzyme. Empty diamonds track the CO peak (1966.6 cm^{-1}) of *Cs*C*c*O solubilized in DDM and empty circles (1966.0 cm^{-1}) refer to the enzyme reconstituted in nanodiscs with *E. coli* polar lipids. The traces are fit with either a single exponential or with the sum of 2 exponentials, respectively. The first time constants are indicated.

To investigate this, we recorded time-resolved difference spectra of *Cs*C*c*O upon CO photolysis from the **R_2CO** under various conditions: In D_2O buffer and with nanodiscs containing ^{13}C-DPPC lipids (as presented in the main text), in H_2O buffer (Tris-HCl, pH 8.0, 5 mM), and in detergent-solubilized *Cs*C*c*O following the protocol previously published by Nyquist et al. [4] (**Figure S2**). The buffer conditions were not kept identical between reconstituted and solubilized samples, in an attempt to better match the experimental conditions used in previous studies [4].

Datasets from nanodisc-reconstituted samples were recorded at ambient temperature instead of 5°C, since there was no need to slow the reaction: *Cs*C*c*O in a membrane environment exhibits a slower proton uptake rate than when detergent-solubilized [1]. Additionally, the rapid scan spectra were complemented with sub-µs-resolved QCL maps.

Comparison of the spectra in **Figure S2** reveals clear differences in the lineshape and peak positions of bands associated with E286. All peak positions shift by ~5 cm^{-1} upon H/D exchange, which is in line with the expected behavior for states in which E286 is hydrogen-bonded to the water wire connecting to the D pathway [5]. However, while the spectra of the reconstituted enzyme show only a negative peak, *Cs*C*c*O in detergent appears to exhibit a blue-shifted negative peak along with a possible positive contribution at frequencies 9 cm^{-1} lower.

Assessing the relative intensity of these peaks is challenging, but their positions and H/D-induced shifts seem to indicate that the reaction of detergent-solubilized and reconstituted enzymes significantly differs between each other. Additional spectral differences are observed in the amide I frequency range; However, given the high optical density of our samples in H_2O, we refrain from interpreting changes within the 1660−1620 cm^{-1} range.

Our observations support the hypothesis that solubilization with Dodecyl-β-D-maltoside significantly alters the reaction of *Cs*C*c*O, inhibiting (at least partially) proton release from E286 and secondary structure changes that are crucially linked in the catalytic cycle. It remains unclear whether these effects arise solely from pK_a shifts, restricted solvent accessibility, increased surface rigidity, or a combination of these factors.

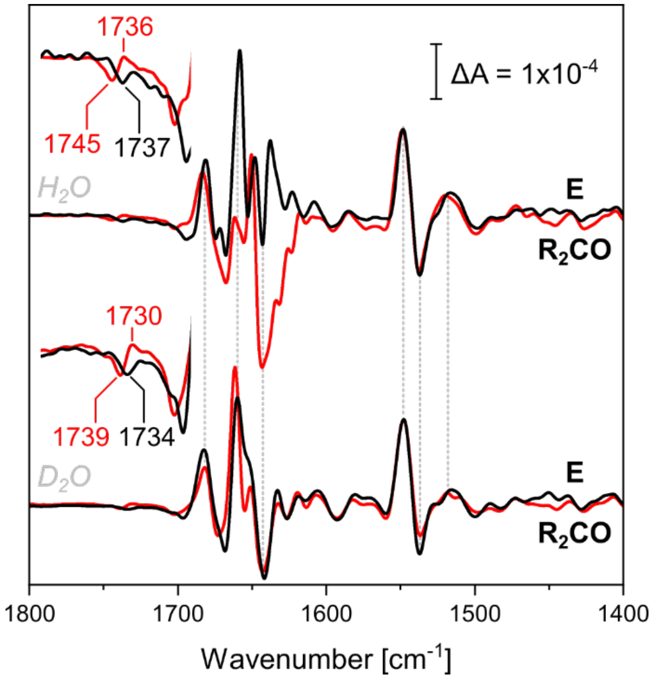

Figure S2. Rapid-scan difference spectra recorded between 0 and 20 ms upon photolysis of the **R_2CO** state of *Cs*C*c*O. The spectra are recorded either in H_2O (top) or D_2O (bottom). Black lines refer to the enzyme reconstituted in ^{13}C-DPPC nanodiscs with Tris/HCl buffer at pH 8.0 and pH*8.2 (22°C), while red lines refer to the enzyme solubilized in DDM and in phosphate buffer at pH 8.5 and pH*8.7 (5°C). The insets show a magnified view of the same spectra.

2. Monitoring the preparation of thin-film samples

Given our preparation protocol, it was important to check the successful preparation of the desired ground states. As mentioned in the main text, one way to do this is to acquire UV/Vis absorbance spectra of the sample upon exposure to carbon monoxide in the presence or absence of $Na_2S_2O_4$. However, the Soret band of C*c*O's heme cofactors is broad, and it is therefore difficult to precisely determine the peak components that are present in a spectrum. Often enough, this problem is circumvented by analyzing the less intense Q-bands in the wavelengths between 500–650 nm. It is known that the Fe-C≡O stretching band of the bound ligand can also be used to determine different redox states of the cofactors, exploiting the vibrational Stark effect [6-8].

We measured FTIR spectra at a resolution of 1 cm^{-1} in the frequency regime of 1950–1975 cm^{-1} before and after photolysis of the **R_2CO** and **R_4CO** samples of *Cs*C*c*O reconstituted in nanodiscs containing ^{13}C-DPPC lipids (**Figure S3**). These three samples resulted in: (I) The IR kinetics presented in panel D of **Figure 2**, and panels A and B of **Figure 4** in the main text; (II) The IR difference absorbance "map" presented in panel A of **Figure S5**, from which we extracted the spectra shown in panel A of **Figure 3** in the main text; (III) The IR kinetics measured upon photolysis of the fully reduced, CO-bound state **R_4CO**, which were used as a control for panel A of **Figure 4** in the main text.

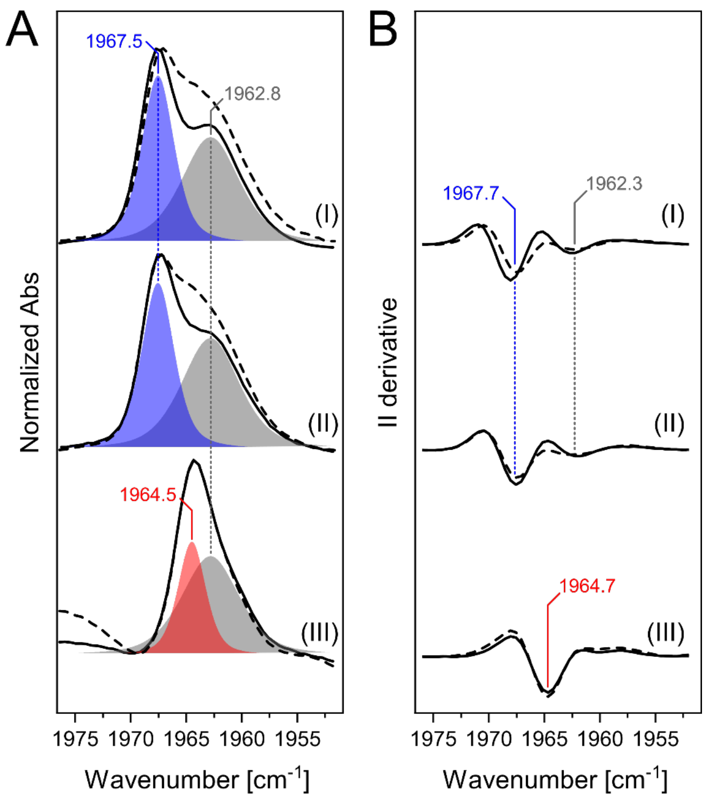

Figure S3: Absolute IR absorption spectra before and after photolysis experiments. (A) From top to bottom: (I) ν(Fe-C≡O) band of the sample used for all QCL kinetics referring to **R_2CO** as a ground state; (II) ν(Fe-C≡O) band of the sample used for all QCL maps recorded upon photolysis of **R_2CO**, presented in the main text as well as in **Figure S5**; (III) ν(Fe-C≡O) band of the fully-reduced sample used as a control in **Fig. 4A** of the main text. The spectra before photolysis were fit to the sum of two Voigtian profiles, whose components are shown as colored shaded areas. Peak positions of the gaussian fit components are labeled. (B) Second derivative of the absolute spectra after smoothing with a 25 point moving average. Peak minima are indicated. In both panels, solid lines refer to the sample before photolysis, while dashed lines refer to the sample after the experiment.

The steady-state IR absorption spectra were baseline-corrected by subtracting a baseline derived from asymmetrical least-square smoothing and, subsequently, fit with the sum of two Voigtians (convolution of a Lorentzian and a Gaussian function) with a Lorentzian/Gaussian ratio of ½. The peak position for the component at 1962.8 cm^{-1} was determined by globally fitting over the three spectra measured before the experiment (**Figure S3A**). The peak at 1967.5 cm^{-1} was also fit over the spectra belonging to **R_2CO** preparations, namely the solid lines labeled (I) and (II). The component at 1964.5 cm^{-1} was determined by fitting only the pre-photolysis spectrum of **R_4CO** (III). Our analysis indicates that the component at 1962.8 cm^{-1} is a feature of all CO bands present in "absolute" absorption spectra, and that its population relative to the component at 1967.5 cm^{-1} increases during the course of the experiment, while its population relative to the component at 1964.5 cm^{-1} remains unaltered.

Directly analyzing the second derivative spectra (**Figure S3B**) reveals maximal curvature at positions very similar to what was derived from the fit, except for the red-shifted component at ~1962.2 cm^{-1}, which is not clearly observable in (III). Only one minimum at 1964.7 cm^{-1} is evident from the second derivative spectrum of **R_4CO**, likely because of the greater overlap integral between the two components.

3. Heat artifact removal and data analysis for time-resolved QCL datasets

Thermal refractive index changes in D_2O are less pronounced than in H_2O within the 1800–1650 cm^{-1} spectral range, since the ν_2 bending mode of D_2O is shifted to 1209 cm^{-1} [9]. However, transient local heating effects can still introduce artifacts in time-resolved IR measurements, particularly for bands with low absorbance.

To quantify this effect under relevant experimental conditions, we employed myoglobin as a model chromophore, acting as a black-body absorber for the 532 nm excitation pulse of the 2nd harmonic output of a Nd:YAG laser. To approximate the 532 nm absorption of a ~400 µM mixed-valence C*c*O sample, we prepared a 1 mM horse heart myoglobin solution, reduced with ~4 equivalents of $Na_2S_2O_4$. The protein was solubilized in buffer (5 mM Tris-HCl, adjusted to pH 8.0 or pH* 8.2 in H_2O or D_2O) and sealed between two BaF_2 windows using a 25 µm spacer adhered with silicone grease.

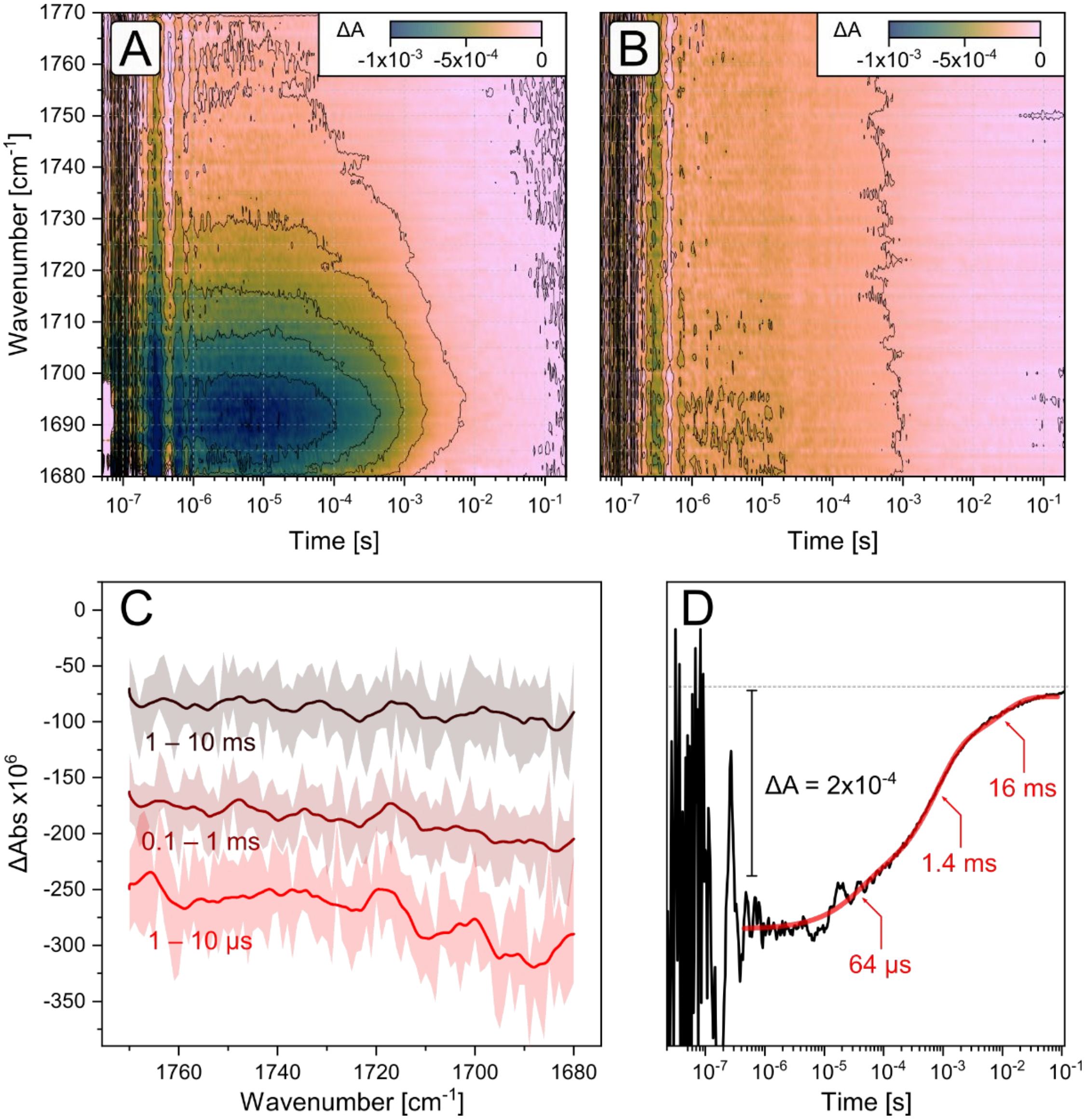

Figure S4: Transient water heating artifacts. Time-resolved IR absorption changes of reduced horse heart myoglobin upon excitation with a 532 nm laser pulse in H_2O (A) or D_2O (B). (C) Selected time-resolved infrared spectra from panel B (D_2O heating). The spectra are averaged between the specified times and their standard deviation is shown as shaded areas. (D) Average of all kinetics in panel B (1680—1770 cm^{-1}). The signal was fitted with the sum of three exponential functions (red line), whose time constants are indicated by red arrows.

The sample was subjected to 32760 laser pulses with an energy density of 20 mJ/cm^2. A forward-backward QCL frequency scan with a step size of 1 cm^{-1} was completed, totaling 360 averaged coadditions for the kinetics corresponding to every step. The scanned frequencies correspond to those presented in the main text. As shown in the IR difference absorbance maps in the carboxyl frequency range (**Figure S4A**-**B**), no narrow spectral features attributable to Mb are identifiable over the noise level, indicating that the protein acts solely as a black body absorber for the laser pulse, increasing its temperature and locally heating the surrounding H_2O or D_2O buffer. Notably, the dataset relative to Mb in D_2O exhibits no detectable spectral features. Averaging the traces across all scanned frequencies in D_2O yields the kinetic profile presented in panel D of **Figure S4**.

While D_2O does not significantly affect time-resolved spectra in the 1680–1770 cm^{-1} range, solvent thermalization can influence the measured kinetics, reaching transient absorbance intensities lower than -2x10^{-4}. Protein-specific kinetics of similar intensity must be corrected for this effect even in D_2O.

The presence of heating artifacts upon excitation of absorbers with 532 nm pulsed Nd-YAG lasers is not an unknown problem. A common approach to mitigate this effect involves intrinsic signal subtraction, where the laser power is increase beyond the reaction saturation point, leaving only a residual thermal signal that scales linearly with power [10]. However, this method is not viable for C*c*O, at least when prepared in the **R₂CO** state. The mixed-valence form of C*c*O is inherently unstable and susceptible to photoreduction when high laser power is used. Reconstitution into lipidic nanodiscs appears to exacerbate this issue, effectively preventing power titrations on the sample used for kinetic measurements, as this would compromise valuable coadditions.

Ultimately, we implemented a data correction procedure that is justified when using C*c*O and targeting the photolysis reaction of its CO-bound states. This approach involves the straightforward subtraction of a reference signal (measured at 1742 cm^{-1}) from the kinetics requiring correction, as described in the main text. The reference signal must be carefully selected to isolate contributions from D_2O heating, ensuring that no other spectral features interfere with the correction.

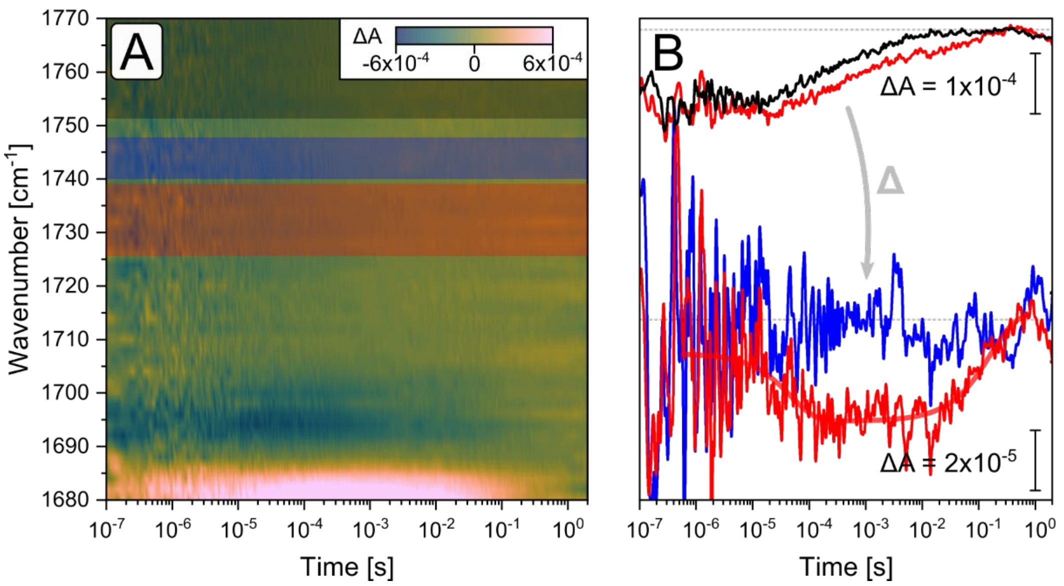

Figure S5: D_2O heat artifact removal from an IR difference absorbance "map". (A) Time-resolved IR absorption changes of the **R_2CO** state of *Cs*C*c*O reconstituted in ^{13}C-DPPC nanodiscs upon photolysis of the CO ligand. The areas shaded in black, blue, and red show the average classes that are used in data analysis for the intrinsic heat artifact removal. (B) Intrinsic heat artifact subtraction procedure: The kinetics corresponding to the black and red shaded areas in A are shown at the top with solid black and solid red lines, respectively. The top traces are subtracted and result in the bottom kinetics (red), which shows the corrected kinetics of the band around 1734 cm^{-1}; A red biexponential fit is overlaid to these kinetics as a guide to the eye. Control kinetics are calculated between the black and blue shaded areas in A and shown at the bottom in blue.

Since *Cs*C*c*O exhibits bands at ~1742 cm^{-1} during some of its reactions [5, 11], using the time-resolved absorbance kinetics recorded at this frequency as a reference raises the question of whether this approach is valid. To demonstrate the reliability of our correction method, we performed an alternative validation: **Figure S5** shows that subtracting the averaged kinetics from the 1752−1770 cm^{-1} region (shaded grey area in **Figure S5A**), in which no transient bands are observed for C*c*O, reproduces the results presented in the main text (**Figure S5B**).

Specifically, subtracting this reference from the averaged kinetics highlighted in blue and red results in two expected outcomes: (**I**) Kinetics at 1740 cm^{-1} that show only control noise, confirming that no relevant transient occurs at this frequency, and (**II**) corrected kinetics at 1734 cm^{-1} that closely match the one presented in **Figure 4** of the main text. We did not apply any rescaling factor to the transients used for subtraction, as panel C in **Figure S4** demonstrates that the heating artifact in D_2O manifests as a nearly constant offset between 1780 and 1680 cm^{-1}, making further adjustments unnecessary.

References

[1] L. N. Öjemyr, C. von Ballmoos, K. Faxén, E. Svahn, P. Brzezinski, *Biochem* **2012**, *51*, 1092-1100.
[2] P. Brzezinski, B. G. Malmström, *FEBS Lett* **1985**, *187*, 111-114.
[3] F. Baserga, J. Storm, R. Schlesinger, J. Heberle, S. T. Stripp, *Biochim Biophys Acta Bioenerg* **2023**, *1864*, 149000.
[4] R. M. Nyquist, D. Heitbrink, C. Bolwien, R. B. Gennis, J. Heberle, *Proc Natl Acad Sci USA* **2003**, *100*, 8715.
[5] R. M. Nyquist, D. Heitbrink, C. Bolwien, T. A. Wells, R. B. Gennis, J. Heberle, *FEBS Lett* **2001**, *505*, 63-67.
[6] E. S. Park, S. S. Andrews, R. B. Hu, S. G. Boxer, *J Phys Chem B* **1999**, *103*, 9813-9817.
[7] E. D. Dodson, X.-J. Zhao, W. Caughey, M. C. Elliott, *Biochemistry* **1996**, *35*, 444-452.
[8] F. Baserga, J. Dragelj, J. Kozuch, H. Mohrmann, E.-W. Knapp, S. T. Stripp, J. Heberle, *Front Chem* **2021**, *9*, 669452.
[9] J.-J. Max, C. Chapados, *J Chem Phys* **2009**, *131*, 184505.
[10] S. M. Mäusle, A. Abzaliyeva, P. Greife, P. S. Simon, R. Perez, Y. Zilliges, H. Dau, *J Chem Phys* **2020**, *153*, 215101.
[11] P. Hellwig, J. Behr, C. Ostermeier, O.-M. H. Richter, U. Pfitzner, A. Odenwald, B. Ludwig, H. Michel, W. Mäntele, *Biochemistry* **1998**, *37*, 7390-7399.
